# Chronic Pain Among Adults — United States, 2019–2021

**DOI:** 10.15585/mmwr.mm7215a1

**Published:** 2023-04-14

**Authors:** S. Michaela Rikard, Andrea E. Strahan, Kristine M. Schmit, Gery P. Guy

**Affiliations:** 1 Division of Overdose Prevention, National Center for Injury Prevention and Control, CDC.

Chronic pain (i.e., pain lasting ≥3 months) is a debilitating condition that affects daily work and life activities for many adults in the United States and has been linked with depression (*1*), Alzheimer disease and related dementias (*2*), higher suicide risk (*3*), and substance use and misuse (*4*). During 2016, an estimated 50 million adults in the United States experienced chronic pain, resulting in substantial health care costs and lost productivity (*5*,*6*). Addressing chronic pain and improving the lives of persons living with pain is a public health imperative. Population research objectives in the National Pain Strategy, which was released in 2016 by the Interagency Pain Research Coordinating Committee, call for more precise estimates of the prevalence of chronic pain and high-impact chronic pain (i.e., chronic pain that results in substantial restriction to daily activities) in the general population and within various population groups to guide efforts to reduce the impact of chronic pain (*3*). Further, a 2022 review of U.S. chronic pain surveillance systems identified the National Health Interview Survey (NHIS) as the best source for pain surveillance data (*7*). CDC analyzed data from the 2019–2021 NHIS to provide updated estimates of the prevalence of chronic pain and high-impact chronic pain among adults in the United States and within population groups defined by demographic, geographic, socioeconomic, and health status characteristics. During 2021, an estimated 20.9% of U.S. adults (51.6 million persons) experienced chronic pain, and 6.9% (17.1 million persons) experienced high-impact chronic pain. New findings from this analysis include that non-Hispanic American Indian or Alaska Native (AI/AN) adults, adults identifying as bisexual, and adults who are divorced or separated are among the populations experiencing a higher prevalence of chronic pain and high-impact chronic pain. Clinicians, practices, health systems, and payers should vigilantly attend to health inequities and ensure access to appropriate, affordable, diversified, coordinated, and effective pain management care for all persons (*8*).

NHIS is a cross-sectional, household survey of the civilian, noninstitutionalized population conducted annually by the National Center for Health Statistics (NCHS).* Data were analyzed using the 2019–2021 Sample Adult Interviews.^†^ Survey questions used to estimate the prevalence of pain included, “In the past three months, how often did you have pain? Would you say never, some days, most days, or every day?” and “Over the past three months, how often did your pain limit your life or work activities? Would you say never, some days, most days, or every day?” If the participant was physically or mentally unable to respond to survey questions, then a knowledgeable proxy was permitted to answer on their behalf. Consistent with previous work, chronic pain was defined as pain on most days or every day during the previous 3 months, and high-impact chronic pain was defined as chronic pain that also limited daily life or work activities on most days or every day during the previous 3 months (*5*).

Using data from the 2019–2021 NHIS, the prevalence of chronic pain and high-impact chronic pain (including estimated number, crude rates, and age-adjusted rates with Korn-Graubard 95% CIs) were estimated for the adult U.S. population overall and within population groups defined by demographic, geographic, socioeconomic, and health status characteristics. Age-adjusted rates were calculated because the prevalence of pain is reported to vary by age (*5*). Estimates not meeting the NCHS reliability standards^§^ were not reported. Demographic characteristics included sex, age, race and ethnicity, sexual orientation, marital status, veteran status, and nativity (U.S.-born or non–U.S.-born). Geographic characteristics included region^¶^ and urban-rural classification. Socioeconomic characteristics included family income relative to the federal poverty level, education level, employment status, and health insurance coverage (reported separately by NHIS for adults aged <65 years and ≥65 years). Health status characteristics included general health status, disability status, and history of chronic medical conditions. Analysis was conducted using SAS (version 9.4; SAS Institute) and used weight and variance estimation variables** that account for the complex survey design of NHIS. All reported differences between subgroups in crude rates and in age-adjusted rates were significantly different on the basis of two-tailed Z-tests (p<0.05). This activity was reviewed by CDC and was conducted consistent with applicable federal law and CDC policy.^††^

Between 2019 and 2021, the prevalence of chronic pain among U.S. adults ranged from 20.5% to 21.8%, and the prevalence of high-impact chronic pain ranged from 6.9% to 7.8% (Figure). During 2021, an estimated 51.6 million U.S. adults (20.9%) experienced chronic pain, and 17.1 million (6.9%) experienced high-impact chronic pain (Table). The age-adjusted prevalence of both chronic pain and high-impact chronic pain was notably higher among certain demographic population groups including AI/AN adults, adults identifying as bisexual, and adults who were divorced or separated. The age-adjusted prevalence of high-impact chronic pain among AI/AN adults (12.8%) was six times as high as among non-Hispanic Asian adults (2.1%) and nearly twice as high as among non-Hispanic White adults (6.5%). The age-adjusted prevalence of chronic pain among adults identifying as bisexual was 32.9%, compared with 19.3% among adults identifying as straight and 20.7% among those identifying as gay or lesbian. The age-adjusted prevalence of chronic pain and high-impact chronic pain among adults who were divorced or separated (29.6% and 10.1%, respectively) was nearly twice as high as among those who were married (18.2% and 5.2%, respectively). The age-adjusted prevalence of chronic pain and high-impact chronic pain among those born in the United States (21.6% and 7.0%, respectively) was nearly twice as high as among those born outside the United States (11.9% and 4.1%, respectively).

**FIGURE:**
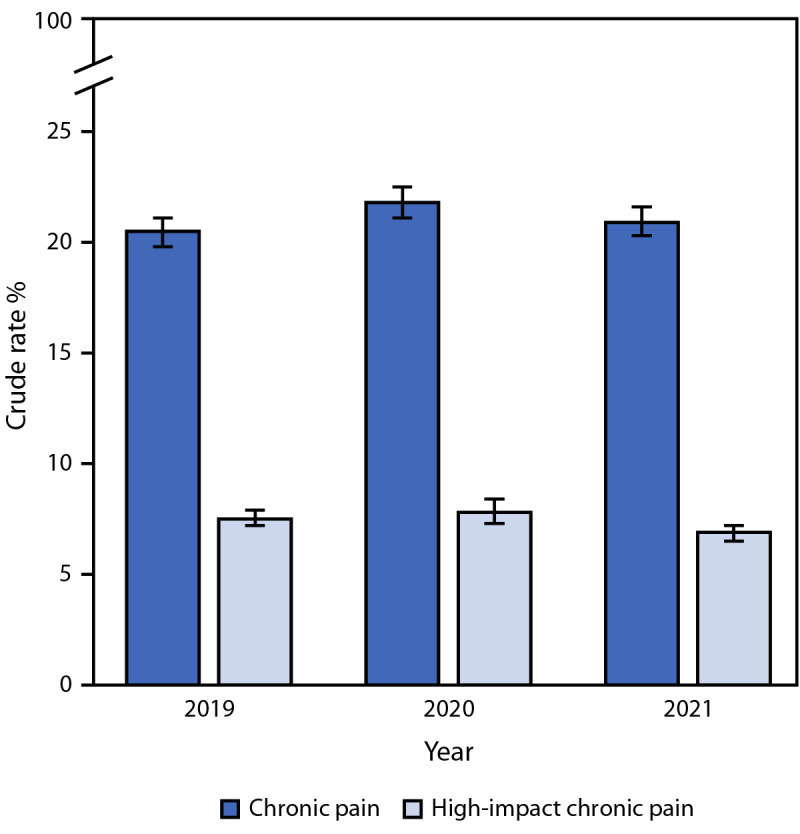
Prevalence of chronic pain* and high-impact chronic pain^†^ among adults — United States, 2019–2021^§^^,^^¶^ * Pain reported on most days or every day during the previous 3 months. ^†^ Chronic pain that limited life or work activities on most days or every day during the previous 3 months. ^§^ Sample sizes for chronic pain and high-impact chronic pain were as follows: 2019 (n = 31,304; n = 31,281), 2020 (n = 31,126; n = 17,409), 2021 (n = 28,759; n = 28,740). Survey responses coded as “refused,” “don’t know,” “not ascertained,” or missing responses were excluded from the analysis. In 2020, the survey question used to define high-impact chronic pain was only included in quarters 3 and 4. Therefore, the sample size was smaller, and survey weights were doubled to produce annual estimates for high-impact chronic pain for 2020. ^¶^ With 95% CIs indicated with error bars.

**TABLE:** Prevalence of chronic pain* and high-impact chronic pain^†^ among adults, by demographic, geographic, socioeconomic, and health status characteristics — United States, 2021

Characteristic	Chronic pain			High-impact chronic pain		
	Estimated no.^§^	Crude rate % (95% CI)	Age-adjusted rate^¶^ % (95% CI)	Estimated no.^§^	Crude rate % (95% CI)	Age-adjusted rate^¶^ % (95% CI)
**Overall**	**51,561,000**	**20.9 (20.2–21.5)**	**19.7 (19.1–20.3)**	**17,100,000**	**6.9 (6.6–7.3)**	**6.4 (6.1–6.7)**
**Sex**						
Female	28,074,000	22.0 (21.2–22.9)	20.5 (19.7–21.3)	9,739,000	7.6 (7.2–8.2)	7.0 (6.5–7.5)
Male	23,484,000	19.7 (18.8–20.5)	18.8 (18.0–19.6)	7,360,000	6.2 (5.7–6.7)	5.8 (5.3–6.2)
**Age group, yrs**						
18–24	2,135,000	7.5 (6.3–9.0)	NA	357,000	1.3 (0.7–2.0)	NA
25–44	11,532,000	13.7 (12.8–14.6)	NA	2,928,000	3.5 (3.0–4.0)	NA
45–64	21,236,000	26.8 (25.7–27.9)	NA	7,938,000	10.0 (9.3–10.8)	NA
65–84	14,694,000	30.0 (28.8–31.3)	NA	5,067,000	10.4 (9.6–11.2)	NA
≥85	1,894,000	34.3 (30.6–38.2)	NA	786,000	14.3 (11.8–17.2)	NA
Race and ethnicity**						
AI/AN, non-Hispanic	991,000	29.8 (22.4–38.0)	28.0 (21.5–34.6)	451,000	13.6 (9.2–19.1)	12.8 (8.6–17.0)
Asian, non-Hispanic	1,136,000	7.8 (6.4–9.3)	7.7 (6.3–9.0)	305,000	2.1 (1.4–3.0)	2.1 (1.4–2.9)
Black or African American, non-Hispanic	5,352,000	18.8 (17.3–20.4)	18.2 (16.7–19.7)	2,247,000	7.9 (6.8–9.1)	7.6 (6.5–8.7)
White, non-Hispanic	37,105,000	23.8 (23.0–24.6)	21.8 (21.0–22.5)	11,631,000	7.5 (7.0–7.9)	6.5 (6.1–6.9)
Hispanic or Latino	6,379,000	15.4 (14.0–16.9)	16.5 (15.0–18.0)	2,191,000	5.3 (4.5–6.1)	5.7 (4.9–6.6)
Other single and multiple race	599,000	18.5 (14.0–23.7)	20.9 (15.8–26.1)	274,000	8.5 (5.2–12.8)	10.5 (6.5–14.5)
**Sexual orientation**						
Straight	47,580,000	20.8 (20.2–21.5)	19.3 (18.7–20.0)	15,772,000	6.9 (6.5–7.3)	6.3 (5.9–6.6)
Gay or lesbian	960,000	19.2 (15.9–22.9)	20.7 (17.3–24.1)	332,000	6.7 (4.6–9.2)	7.0 (4.8–9.2)
Bisexual	1,384,000	24.3 (20.2–28.7)	32.9 (27.7–38.1)	357,000	6.3 (4.4–8.7)	9.9 (6.6–13.1)
**Marital status**						
Married	26,750,000	21.1 (20.3–22.0)	18.2 (17.3–19.2)	8,079,000	6.4 (5.9–6.9)	5.2 (4.7–5.6)
Widowed	4,673,000	32.9 (30.7–35.1)	25.9 (19.9–31.9)	1,836,000	12.9 (11.5–14.5)	8.1 (5.8–10.3)
Divorced or separated	7,820,000	32.1 (30.5–33.8)	29.6 (24.5–34.6)	3,214,000	13.2 (12.0–14.5)	10.1 (8.9–11.3)
Never married	7,596,000	13.0 (12.0–14.1)	19.2 (17.8–20.5)	2,339,000	4.0 (3.5–4.6)	6.8 (5.9–7.7)
Living with a partner	4,197,000	20.2 (18.1–22.4)	22.9 (20.6–25.1)	1,443,000	6.9 (5.7–8.3)	8.5 (7.0–10.1)
**Veteran status**						
Veteran	5,915,000	32.0 (29.9–34.1)	27.5 (24.9–30.1)	2,132,000	11.6 (10.2–13.0)	9.1 (7.6–10.5)
Not veteran	45,170,000	20.0 (19.3–20.6)	19.2 (18.6–19.8)	14,788,000	6.5 (6.2–6.9)	6.2 (5.9–6.6)
**Nativity**						
U.S.-born	45,340,000	22.7 (22.0–23.4)	21.6 (20.9–22.2)	14,897,000	7.5 (7.1–7.9)	7.0 (6.6–7.4)
Non–U.S.-born	5,759,000	12.8 (11.6–14.1)	11.9 (10.8–13.1)	2,028,000	4.5 (3.9–5.3)	4.1 (3.5–4.8)
**U.S. Census Bureau region**						
Northeast	7,796,000	18.2 (16.7–19.7)	16.8 (15.5–18.3)	2,309,000	5.4 (4.6–6.3)	4.9 (4.1–5.8)
Midwest	11,526,000	22.3 (20.9–23.7)	20.9 (19.7–22.2)	3,514,000	6.8 (6.1–7.5)	6.2 (5.5–6.8)
South	20,337,000	21.8 (20.7–22.8)	20.4 (19.4–21.4)	7,352,000	7.9 (7.3–8.5)	7.2 (6.6–7.8)
West	11,903,000	20.2 (18.9–21.6)	19.6 (18.4–20.8)	3,926,000	6.7 (5.9–7.5)	6.4 (5.7–7.1)
**Urban-rural classification** ^††^						
Large central metro	13,387,000	17.1 (16.1–18.2)	16.8 (15.8–17.8)	4,433,000	5.7 (5.1–6.4)	5.5 (4.9–6.2)
Large fringe metro	11,073,000	18.7 (17.6–19.9)	17.4 (16.3–18.5)	3,385,000	5.7 (5.1–6.4)	5.2 (4.6–5.8)
Medium and small metro	17,868,000	23.4 (22.1–24.7)	21.9 (20.8–23.1)	5,780,000	7.6 (6.9–8.3)	6.9 (6.3–7.6)
Nonmetropolitan	9,233,000	27.8 (25.7–30.0)	25.4 (23.5–27.4)	3,502,000	10.6 (9.4–11.8)	9.2 (8.2–10.3)
**Family income** ^§§^						
<100% FPL	6,921,000	28.5 (26.3–30.9)	28.8 (26.6–31.0)	3,444,000	14.2 (12.7–15.9)	14.4 (12.8–16.0)
100% to <200% FPL	10,803,000	25.1 (23.6–26.6)	24.1 (22.6–25.6)	4,220,000	9.8 (8.9–10.8)	9.5 (8.6–10.5)
200% to <400% FPL	15,983,000	22.0 (20.9–23.1)	20.9 (19.9–22.0)	5,248,000	7.2 (6.6–7.9)	6.7 (6.1–7.3)
≥400% FPL	17,855,000	16.7 (16.0–17.5)	15.3 (14.6–16.1)	4,187,000	3.9 (3.5–4.3)	3.5 (3.1–3.9)
**Education**						
No high school diploma or GED	5,898,000	25.7 (23.6–27.9)	22.6 (20.5–24.8)	2,464,000	10.8 (9.5–12.1)	8.9 (7.6–10.1)
High school diploma or GED	15,615,000	22.5 (21.3–23.7)	21.7 (20.6–22.9)	5,756,000	8.3 (7.6–9.1)	7.9 (7.2–8.6)
Some college	15,979,000	24.5 (23.3–25.7)	24.2 (23.0–25.3)	5,441,000	8.3 (7.6–9.1)	8.1 (7.4–8.9)
Bachelor’s degree or higher	13,804,000	15.7 (15.0–16.5)	14.8 (14.0–15.6)	3,291,000	3.8 (3.4–4.2)	3.4 (3.0–3.7)
**Employment status**						
Employed^¶¶^	24,045,000	15.8 (15.1–16.6)	16.5 (15.7–17.3)	5,088,000	3.4 (3.0–3.7)	3.7 (3.2–4.1)
Not employed, worked previously	26,093,000	30.5 (29.4–31.6)	26.7 (25.5–28.0)	11,461,000	13.4 (12.6–14.2)	12.7 (11.8–13.7)
Not employed, never worked	822,000	13.1 (10.2–16.3)	15.6 (12.3–19.0)	334,000	5.3 (3.5–7.6)	6.8 (4.3–9.3)
Health insurance coverage***						
**Aged <65 yrs**						
Private	21,401,000	16.1 (15.4–16.9)	15.3 (14.6–16.0)	5,348,000	4.0 (3.6–4.5)	3.7 (3.3–4.1)
Medicaid and other public coverage	6,899,000	25.7 (23.7–27.8)	26.9 (25.0–28.8)	3,295,000	12.3 (10.9–13.8)	13.1 (11.7–14.4)
Other coverage	2,999,000	38.6 (35.1–42.2)	32.5 (28.9–36.0)	1,589,000	20.5 (17.6–23.6)	16.9 (13.8–20.0)
Uninsured	3,532,000	14.7 (12.9–16.5)	14.9 (13.1–16.7)	972,000	4.0 (3.2–5.1)	4.2 (3.2–5.1)
**Aged ≥65 yrs**						
Private	5,938,000	28.8 (27.0–30.7)	29.1 (27.3–30.9)	1,734,000	8.4 (7.4–9.5)	8.6 (7.6–9.6)
Medicare and Medicaid	1,595,000	43.1 (37.7–48.5)	43.1 (37.8–48.4)	800,000	21.8 (17.8–26.3)	21.8 (17.7–25.9)
Medicare Advantage	5,574,000	29.8 (27.8–31.8)	29.9 (27.9–31.8)	2,005,000	10.7 (9.5–12.1)	10.7 (9.4–12.0)
Medicare only, excluding Medicare Advantage	1,810,000	27.1 (23.9–30.6)	27.2 (23.9–30.5)	646,000	9.7 (7.7–12.0)	9.8 (7.7–11.9)
Other coverage	1,624,000	35.8 (31.7–40.1)	35.5 (31.4–39.6)	669,000	14.7 (11.9–18.0)	14.8 (11.8–17.8)
Uninsured	53,000	—^†††^	—^†††^	9,000	—^†††^	—^†††^
**General health status**						
Excellent	4,221,000	7.0 (6.2–7.7)	7.4 (6.7–8.2)	666,000	1.1 (0.8–1.5)	1.2 (0.9–1.5)
Very good	12,421,000	14.7 (13.9–15.6)	14.3 (13.5–15.1)	2,063,000	2.4 (2.1–2.8)	2.3 (2.0–2.7)
Good	17,659,000	25.8 (24.6–27.0)	23.5 (22.3–24.6)	5,095,000	7.4 (6.8–8.1)	6.5 (5.9–7.1)
Fair	12,016,000	47.0 (44.8–49.4)	42.5 (39.6–45.4)	5,684,000	22.3 (20.6–24.1)	19.5 (17.5–21.4)
Poor	5,212,000	69.2 (65.4–72.9)	67.6 (60.5–74.7)	3,578,000	48.3 (44.4–52.2)	48.7 (41.8–55.7)
**Disability status** ^§§§^						
With disability	12,489,000	58.0 (55.7–60.3)	52.4 (49.3–55.5)	7,391,000	34.5 (32.5–36.5)	32.0 (29.3–34.8)
Without disability	39,072,000	17.3 (16.7–17.9)	16.8 (16.2–17.4)	9,709,000	4.3 (4.0–4.6)	4.1 (3.8–4.4)
**Chronic medical conditions** ^¶¶¶^						
**Cardiovascular**						
Hypertension	25,625,000	33.0 (31.9–34.2)	27.3 (25.7–28.9)	10,268,000	13.2 (12.5–14.1)	10.6 (9.7–11.5)
High cholesterol	21,012,000	31.6 (30.3–32.9)	25.2 (23.6–26.8)	7,905,000	11.9 (11.1–12.8)	9.0 (8.1–9.9)
Coronary heart disease	4,977,000	40.7 (37.9–43.6)	33.8 (25.4–42.2)	2,382,000	19.5 (17.3–21.9)	16.6 (11.2–22.0)
Angina	1,906,000	50.9 (45.6–56.3)	44.0 (34.6–53.3)	1,122,000	30.0 (25.2–35.1)	27.7 (19.3–36.2)
Myocardial infarction	3,319,000	44.0 (40.2–47.8)	40.3 (31.1–49.5)	1,615,000	21.5 (18.6–24.5)	17.1 (11.8–22.4)
Stroke	3,176,000	46.0 (42.2–49.9)	43.4 (34.8–52.0)	1,692,000	24.8 (21.6–28.3)	23.8 (17.9–29.7)
**Respiratory**						
COPD, emphysema, or chronic bronchitis	6,210,000	54.5 (51.6–57.4)	50.0 (44.4–55.5)	3,130,000	27.5 (24.9–30.2)	23.9 (19.8–28.0)
Asthma	6,926,000	35.0 (32.7–37.3)	33.6 (31.4–35.7)	2,964,000	15.0 (13.4–16.7)	14.2 (12.7–15.8)
**Psychiatric**						
Anxiety disorder	15,163,000	37.4 (35.7–39.1)	37.5 (35.9–39.0)	6,596,000	16.3 (15.0–17.6)	16.3 (15.1–17.5)
Depression	16,776,000	39.0 (37.4–40.7)	38.0 (36.4–39.5)	7,535,000	17.6 (16.4–18.8)	16.8 (15.7–17.9)
**Digestive**						
Hepatitis	1,534,000	38.9 (33.7–44.2)	28.2 (23.3–33.2)	740,000	18.8 (14.9–23.1)	12.9 (9.6–16.2)
Cirrhosis or liver condition	1,120,000	50.8 (43.7–57.8)	40.9 (34.4–47.4)	643,000	29.1 (23.4–35.4)	22.3 (17.3–27.3)
**Musculoskeletal**						
Arthritis	26,796,000	51.0 (49.5–52.5)	51.3 (48.3–54.4)	10,700,000	20.4 (19.2–21.6)	20.0 (17.6–22.4)
**Endocrine**						
Diabetes	8,954,000	37.7 (35.5–39.9)	29.1 (26.2–32.0)	3,916,000	16.5 (15.1–18.1)	12.9 (10.9–14.8)
**Neurologic**						
Dementia	1,151,000	44.8 (39.0–50.7)	54.9 (38.4–71.3)	648,000	25.3 (20.2–30.9)	34.2 (19.9–48.5)
Epilepsy	1,868,000	42.4 (37.0–47.9)	40.0 (34.8–45.1)	912,000	20.7 (16.7–25.1)	19.6 (15.7–23.5)
**Genitourinary**						
Chronic kidney disease	3,760,000	53.8 (50.1–57.4)	48.4 (42.0–54.7)	1,954,000	28.0 (24.7–31.5)	25.4 (19.8–31.1)
**Multiple systems**						
Cancer	8,536,000	35.1 (33.3–37.0)	30.6 (26.2–35.0)	3,258,000	13.4 (12.2–14.7)	11.9 (9.9–13.8)
ME/CFS	1,914,000	74.5 (68.5 –79.8)	70.0 (63.1–76.9)	1,242,000	48.3 (42.2–54.4)	43.8 (37.0–50.7)

**Abbreviations:** AI/AN = American Indian or Alaska Native, non-Hispanic; COPD = chronic obstructive pulmonary disease; FPL = federal poverty level; GED = general educational development certificate; ME/CFS = myalgic encephalomyelitis/chronic fatigue syndrome; NA = not applicable; NHIS = National Health Interview Survey.

* Pain reported on most days or every day during the previous 3 months.

^†^ Chronic pain that limits life or work activities on most days or every day during the previous 3 months.

^§^ Weighted estimates are rounded to the nearest 1,000. Responses coded as “refused,” “don’t know,” “not ascertained,” or missing responses were excluded from the analysis.

^¶^ Estimates are age-adjusted using the projected 2000 U.S. Census Bureau population as the standard population and five age groups (18–24, 25–44, 45–64, 65–84, and ≥85 years) except for health insurance coverage. For health insurance coverage for those aged <65 years, three age groups were used (18–24, 25–44, and 45–64), and for health insurance coverage for those aged ≥65 years, two age groups were used (65–84 and ≥85).

** Persons who reported AI/AN only or AI/AN and another race are included in the AI/AN category. Persons who reported a single race other than Black or African American, Asian, AI/AN, or White, or who reported more than one race not including AI/AN were combined into the “other single and multiple race” category.

^††^ Based on the 2013 National Center for Health Statistics Urban-Rural Classification Scheme for Counties (https://www.cdc.gov/nchs/data/series/sr_02/sr02_166.pdf). Nonmetropolitan includes counties in micropolitan statistical areas and noncore counties.

^§§^ Missing data on family income and earnings in the NHIS are imputed using a multiple imputation methodology. Family income is reported as a percentage of the FPL using the weighted average thresholds published annually by the U.S. Census Bureau. https://www.census.gov/data/tables/time-series/demo/income-poverty/historical-poverty-thresholds.html

^¶¶^ Persons who performed seasonal or contract work and worked during the previous 12 months, or those who were working at a job or business, but not for pay, were considered employed.

*** https://ftp.cdc.gov/pub/Health_Statistics/NCHS/Dataset_Documentation/NHIS/2021/srvydesc-508.pdf

^†††^ Estimates considered unreliable according to the National Center for Health Statistics’ standards of reliability. https://www.cdc.gov/nchs/data/series/sr_02/sr02_175.pdf

^§§§^ Based on the Washington Group Adult Composite Disability Indicator.

^¶¶¶^ Based on the survey question, “Have you ever been told by a doctor or other health professional that you had…” except for asthma and ME/CFS, which are a combination of the previous question and “Do you still have asthma?,” or “Do you still have CFS or ME?”

Within population groups defined by geographic and socioeconomic characteristics, the age-adjusted prevalence of high-impact chronic pain among adults residing in nonmetropolitan areas (9.2%) and adults with a family income <100% of the federal poverty level (FPL) (14.4%) was approximately two and four times as high, respectively, as among those residing in large central metro areas (5.5%) and those with family income ≥400% FPL (3.5%). Adults reporting poor general health and adults with a disability experienced an exceptionally high prevalence of chronic pain (67.6% and 52.4%, respectively) and high-impact chronic pain (48.7% and 32.0%, respectively). Among all chronic medical conditions reported, the age-adjusted prevalence of chronic pain and high-impact chronic pain was highest among adults with a history of myalgic encephalomyelitis/chronic fatigue syndrome (70.0% and 43.8%, respectively) and dementia (54.9% and 34.2%, respectively).

## Discussion

Chronic pain is a debilitating condition that affects the lives of millions of adults in the United States. During 2021, an estimated 20.9% of U.S. adults experienced chronic pain, similar to the reported estimate of 20.4% in 2016 (*5*). The estimated prevalence of high-impact chronic pain in 2021 (6.9%) was, however, lower than in 2016 (8.0%) (*5*). Further, the age-adjusted prevalence of high-impact chronic pain in 2021 was 6.4%, which is the goal set by the Healthy People 2030 objective to reduce the prevalence of high-impact chronic pain (*9*).

Findings in this report highlight important disparities in the prevalence of chronic pain among certain population groups. Consistent with previous studies, the prevalences of chronic pain and high-impact chronic pain were higher among older adults, females, adults currently unemployed but who worked previously, veterans, adults living in poverty, those residing in nonmetropolitan areas, and those with public health insurance (*5*). This report contributes additional findings that the prevalences of chronic pain and high-impact chronic pain were also higher among AI/AN adults, adults identifying as bisexual, those who are divorced or separated, U.S.-born adults compared with non–U.S.-born adults, adults with a disability, adults in poor health, and adults with a history of certain chronic medical conditions.

Previous studies have identified disparities in the treatment of chronic pain and access to affordable and effective pain management care, yet further work is needed to understand why these disparities exist and to identify opportunities for appropriate and effective interventions (*10*). CDC’s 2022 Clinical Practice Guideline for Prescribing Opioids for Pain provides recommendations to promote a multimodal and multidisciplinary approach to pain management and implementation strategies to reduce disparities in pain management care (*8*). In addition, policies and programs that address primary injury prevention, improved access to affordable, culturally responsive health care, and more effective pain management therapies can mitigate the burden of chronic pain (*3*).

The findings in this report are subject to at least three limitations. First, the results are generalizable only to the noninstitutionalized, civilian adult population; military personnel and persons in nursing homes and other institutions were excluded. Second, survey responses are self-reported and subject to recall bias. Finally, the COVID-19 pandemic affected the collection of survey responses^§§^ and had impacts on health care access and utilization that might affect these results in unknown ways.

Consistent with the population research objectives of the National Pain Strategy to provide more precise estimates of pain among various population groups, this study provides updated estimates of the prevalence of chronic pain and high-impact chronic pain and highlights disparities in the prevalence of pain among certain populations. These findings can guide policymakers, clinicians, and researchers in future research examining the underlying reasons for disparities and in the development of tailored interventions and strategies addressing chronic pain in the United States.

**Summary**

**What is already known about this topic?**

An estimated 50 million adults in the United States experienced chronic pain (i.e., pain lasting ≥3 months) in 2016, resulting in substantial health care costs and lost productivity.

**What is added by this report?**

During 2021, an estimated 20.9% of U.S. adults (51.6 million persons) experienced chronic pain, and 6.9% (17.1 million persons) experienced high-impact chronic pain (i.e., chronic pain that results in substantial restriction to daily activities) with a higher prevalence among non-Hispanic American Indian or Alaska Native adults, adults identifying as bisexual, and adults who were divorced or separated.

**What are the implications for public health practice?**

Clinicians, practices, health systems, and payers should vigilantly attend to health inequities and ensure access to appropriate, affordable, diversified, coordinated, and effective pain management care for all persons.

## Footnotes

*
https://www.cdc.gov/nchs/nhis/index.htm

† Sample size and response rates for each year of the NHIS were as follows: 2019 (n = 31,997; response rate = 61.1%), 2020 (n = 31,568; response rate = 48.9%), and 2021 (n = 29,482; response rate = 50.9%). More information is available in the NHIS 2019–2021 survey description documents. https://www.cdc.gov/nchs/nhis/data-questionnaires-documentation.htm

§
https://www.cdc.gov/nchs/data/series/sr_02/sr02_175.pdf

¶ States are grouped into four regions used by the U.S. Census Bureau. https://www2.census.gov/geo/pdfs/maps-data/maps/reference/us_regdiv.pdf

** During 2020, the survey question used to define high-impact chronic pain was only included in quarters 3 and 4. Therefore, weights were doubled to produce annual estimates for high-impact chronic pain for the year 2020.

†† 5 C.F.R. part 46, 21 C.F.R. part 56; 42 U.S.C. Sect. 241(d); 5 U.S.C. Sect. 552a; 44 U.S.C. Sect. 3501 et seq.

§§ Typical data collection procedures were disrupted, attributable to the COVID-19 pandemic. Additional documentation regarding changes to data collection procedures is available at the NHIS website. https://www.cdc.gov/nchs/nhis/2020nhisdata.htm
